# Tocilizumab for the treatment of refractory pediatric mixed connective tissue disease (MCTD), in two patients

**DOI:** 10.1186/1546-0096-12-S1-P303

**Published:** 2014-09-17

**Authors:** Natalia Cabrera, Marie Caroline Chastang, Marine Desjonquères, Agnes Duquesne, Pierre Cochat, Alexandre Belot

**Affiliations:** 1 Rheumatology, Nephrology and Pediatric Dermatology., Hospital Femme Mère Enfant, Lyon, France

## Introduction

Mixed Connective Tissue Disease (MCTD) is a rare autoimmune disease of unknown origin. The diagnosis is suggested when there are overlaping symptoms of lupus, scleroderma and dermatomyositis. Kasukawa criteria are the most commonly used in pediatrics. Treatment should be tailored to the clinical symptoms but no specific recommendation regarding the therapeutic management has been established to date.

Tocilizumab (TCZ), an inhibitor of interleukin 6 is used in juvenile idiopathic arthritis (systemic and polyarticular JIA) and Castleman's disease. Only one case has been reported with TCZ in the context of a MCTD with pulmonary hypertension in a 45 year-old man with a favorable outcome.

## Objectives

We report on two children diagnosed for a MCTD who presented with active arthritis despite various therapies including methotrexate (MTX) and TNFa blockers.

## Methods

Description of two clinical cases.

## Results

### Case 1

A 7 year-old girl was initially diagnosed for a polyarticular JIA and was in remission under MTX and etanercept (ETA) combination. 8 years later (at the age of 15), she presented with a relapse with Raynaud's phenomenon, sclerodactily and polyarticular arthritis. Laboratory tests revealed the presence of ANA (1/1600), positive anti-DNAdb (30 UI/l) and positive anti-ENA (anti Sm–RNP at >8 UA/ml and anti U1RNP at >8 UA/ml). Because of low tolerance to MTX and joint manifestations in the foreground, treatment with TCZ was initiated. The joint outcome under treatment was shortly favorable. Regarding other autoimmune manifestations, the evolution was marked by the appearance of positive Coombs test with asymptomatic anemia, neutropenia, mixed, and an increase of anti-DNA titer. Other treatments consisted in a low dose steroids and hydroxychloroquine (HCQ).

### Case 2

A 12 year-old girl presented with juvenile dermatomyositis and was successfully treated by steroids and MTX. 2 years later, she presented a relapse with Raynaud's phenomenon, sclerodactily, and a Gougerot-Sjögren’s syndrome. Autoantibody screening revealed positive ANA (1/1600) with anti-DNA 7,4 (Farr assay) and positive anti-ENA (anti -SSA, anti -Sm, anti- SmRNP and anti- U1RNP). Various therapies including MTX, mycophenolate mofetil, HCQ, ETA did not manage to control articular symptoms. TCZ in combination with MTX was started at the age of 17 and was shortly associated to remission. Other manifestations, including Raynaud's syndrome and liver disease, tended to persist. Other therapies consisted in a low dose steroids and HCQ with irregular compliance.

## Conclusion

This is the first description of the use of TCZ for articular manifestations of pediatric-onset MCTD. These two observations suggest that TCZ is effective and safe on articular manifestations of MCTD. However, systemic autoimmune manifestations including leucopenia and hepatitis were not improved by the treatment.

TCZ is as a therapeutic option in the MCTD with polyarticular manifestations refractory or intolerant to basic treatment.

## Disclosure of interest

None declared.

